# Pre-exposure rabies prophylaxis: a systematic review

**DOI:** 10.2471/BLT.16.173039

**Published:** 2016-11-25

**Authors:** Jocelyn A Kessels, Sergio Recuenco, Ana Maria Navarro-Vela, Raffy Deray, Marco Vigilato, Hildegund Ertl, David Durrheim, Helen Rees, Louis H Nel, Bernadette Abela-Ridder, Deborah Briggs

**Affiliations:** a School of Veterinary Science, University of Queensland Gatton Campus, Via Warrego Highway, Gatton, Queensland 4343, Australia.; b National Centre for Public Health, Instituto Nacional de Salud, Lima, Peru.; c Directorate General of Strategic Interventions in Public Health, Ministry of Health, Lima, Peru.; d Diseases Prevention and Control Bureau, Department of Health, Manilla, Philippines.; e Veterinary Public Health Unit, Pan American Health Organisation–World Health Organisation, Rio de Janeiro, Brazil.; f Wistar Institute Vaccine Center, Philadelphia, United States of America (USA).; g Hunter Medical Research Institution, University of Newcastle, Newcastle, Australia.; h Wits Reproductive Health and HIV Institute, University of Witwatersrand, Johannesburg, South Africa.; i Department of Microbiology and Plant Pathology, University of Pretoria, Pretoria, South Africa.; j Neglected Zoonotic Diseases, World Health Organization, Geneva, Switzerland.; k College of Veterinary Medicine, Kansas State University, Manhattan, USA.

## Abstract

**Objective:**

To review the safety and immunogenicity of pre-exposure rabies prophylaxis (including accelerated schedules, co-administration with other vaccines and booster doses), its cost–effectiveness and recommendations for use, particularly in high-risk settings.

**Methods:**

We searched the PubMed, Centre for Agriculture and Biosciences International, Cochrane Library and Web of Science databases for papers on pre-exposure rabies prophylaxis published between 2007 and 29 January 2016. We reviewed field data from pre-exposure prophylaxis campaigns in Peru and the Philippines.

**Findings:**

Pre-exposure rabies prophylaxis was safe and immunogenic in children and adults, also when co-administered with routine childhood vaccinations and the Japanese encephalitis vaccine. The evidence available indicates that shorter regimens and regimens involving fewer doses are safe and immunogenic and that booster intervals could be extended up to 10 years. The few studies on cost suggest that, at current vaccine and delivery costs, pre-exposure prophylaxis campaigns would not be cost-effective in most situations. Although pre-exposure prophylaxis has been advocated for high-risk populations, only Peru and the Philippines have implemented appropriate national programmes. In the future, accelerated regimens and novel vaccines could simplify delivery and increase affordability.

**Conclusion:**

Pre-exposure rabies prophylaxis is safe and immunogenic and should be considered: (i) where access to postexposure prophylaxis is limited or delayed; (ii) where the risk of exposure is high and may go unrecognized; and (iii) where controlling rabies in the animal reservoir is difficult. Pre-exposure prophylaxis should not distract from canine vaccination efforts, provision of postexposure prophylaxis or education to increase rabies awareness in local communities.

## Résumé

**Objectif:**

Analyser l'innocuité et l'immunogénicité de la prophylaxie pré-exposition à la rage (notamment le schéma de vaccination accéléré, la co-administration d'autres vaccins et les injections de rappel), son rapport coût-efficacité ainsi que les recommandations d'utilisation, en particulier dans les zones à haut risque.

**Méthodes:**

Nous avons recherché, dans les bases de données de PubMed, du Centre for Agriculture and Biosciences International, de la Cochrane Library et de Web of Science, des articles sur la prophylaxie pré-exposition à la rage publiés entre 2007 et le 29 janvier 2016. Nous avons aussi analysé des données de terrain provenant de campagnes pour la prophylaxie pré-exposition menées au Pérou et aux Philippines.

**Résultats:**

La prophylaxie pré-exposition à la rage était sûre et immunogène pour les enfants et les adultes, même co-administrée avec les vaccins systématiques des enfants et le vaccin contre l'encéphalite japonaise. Les éléments disponibles indiquent que les programmes de vaccination plus courts ainsi que ceux comportant des doses plus faibles sont sûrs et immunogènes et que les intervalles de rappel pourraient aller jusqu'à 10 ans. Selon les rares études sur les coûts, en tenant compte du coût actuel des vaccins et de leur administration, dans la plupart des cas, les campagnes pour la prophylaxie pré-exposition ne seraient pas rentables. Même s’il a été recommandé d’appliquer une prophylaxie pré-exposition dans les populations à haut risque, seuls le Pérou et les Philippines ont mis en œuvre des programmes nationaux à cet égard. Dans l'avenir, des schémas de vaccination accélérés et de nouveaux vaccins pourraient en simplifier l'administration, à des prix plus abordables.

**Conclusion:**

La prophylaxie pré-exposition à la rage est sûre et immunogène et devrait être envisagée: (i) lorsque l'accès à la prophylaxie post-exposition est limité ou tardif; (ii) lorsque le risque d'exposition est élevé et pourrait passer inaperçu; et (iii) lorsqu'il est difficile de lutter contre la rage dans le réservoir animal. La prophylaxie pré-exposition ne doit pas empêcher les efforts de vaccination des chiens, la prophylaxie post-exposition ou la sensibilisation à la prévention de la rage dans les communautés locales.

## Resumen

**Objetivo:**

Analizar la seguridad y la inmunogenicidad de la profilaxis pre exposición a la rabia (incluidos programas acelerados, administración conjunta con otras vacunas y dosis de refuerzo), su rentabilidad y las recomendaciones de uso, especialmente en entornos de alto riesgo.

**Métodos:**

Se realizaron búsquedas en PubMed, el Centro Internacional de Agricultura y Ciencias Biológicas, la Biblioteca Cochrane y la base de datos de la Web of Science en busca de documentos sobre la profilaxis pre exposición a la rabia publicados entre 2007 y el 29 de enero de 2016. Se analizaron datos archivados de campañas de profilaxis pre exposición en Filipinas y Perú.

**Resultados:**

La profilaxis pre exposición a la rabia era segura e inmunogénica en niños y adultos, también cuando se administraba en conjunto con vacunas infantiles rutinarias y la vacuna de la encefalitis japonesa. Las pruebas disponibles indican que los regímenes más cortos y los que implican un menor número de dosis son seguros e inmunogénicos, y que los intervalos de refuerzo podrían ampliarse hasta 10 años. Los pocos estudios sobre el coste sugieren que, con los costes actuales de vacunación y suministro, las campañas de profilaxis pre exposición no serían rentables en la mayoría de las situaciones. A pesar de que la profilaxis pre exposición está destinada para poblaciones de alto riesgo, únicamente Filipinas y Perú han implementado los programas nacionales adecuados. En el futuro, los regímenes acelerados y las nuevas vacunas podrían simplificar el suministro y aumentar la asequibilidad.

**Conclusión:**

La profilaxis pre exposición a la rabia es segura e inmunogénica y debe tenerse en cuenta: (i) cuando el acceso a la profilaxis post exposición sea limitada o se retrase; (ii) cuando el riesgo de exposición sea alto y pueda pasar desapercibido; y (iii) cuando sea complicado controlar la rabia en una reserva animal. La profilaxis pre exposición no debe apartar la atención de las vacunas caninas, el suministro de profilaxis post exposición o la educación para aumentar la concienciación sobre la rabia en comunidades locales.

## ملخص

**الغرض:**

مراجعة عوامل الأمان والاستمناع القرينة بإجراءات الوقاية الطبية السابقة على التعرض لمرض السُعار (داء الكلب) (بما يشمل الجداول الزمنية المعجّلة، وتقديم الأدوية مع غيرها من التحصينات والجرعات المنشطة)، وفعالية التكلفة والتوصيات الخاصة بالاستعمال، وخاصةً في البيئات عالية الخطورة.

**الطريقة:**

قمنا بالبحث في قواعد بيانات PubMed، والمركز الدولي للزراعة والعلوم الحيوية، ومكتبة Cochrane وشبكة العلوم لإيجاد دراسات تتعلق بإجراءات الوقاية الطبية السابقة على التعرض لمرض السُعار والتي يعود تاريخ نشرها إلى الفترة ما بين 2007 و29 كانون الثاني/يناير 2016. وقمنا بمراجعة البيانات الميدانية المأخوذة من حملات الوقاية الطبية السابقة على التعرض لمرض السُعار التي تمت في بيرو والفلبين.

**النتائج:**

كانت الوقاية الطبية السابقة على التعرض لمرض السُعار تراعي عوامل الأمان والاستمناع لدى الأطفال والكبار، وعند تقديمها مع التحصينات الروتينية المقدمة في مرحلة الطفولة ومع تحصين التهاب الدماغ الياباني. وتشير الأدلة المتوفرة إلى أن البرامج العلاجية الأقصر والبرامج العلاجية التي تعتمد على تقديم جرعات أقل تراعي عوامل الأمان والاستمناع، وكان من الممكن إطالة الفترات الزمنية ما بين الجرعات المنشطة إلى ما يصل إلى 10 سنوات. وتشير الدراسات القليلة حول التكلفة إلى أن حملات الوقاية الطبية السابقة على التعرض للمرض لن تكون ذات تكلفة فعالة في أغلب المواقف بالنظر إلى النفقات الحالية للتحصينات وتقديمها. وعلى الرغم من الدعوة إلى تقديم الوقاية الطبية السابقة على التعرض للمرض للقطاعات السكانية المعرضة لدرجة عالية من الخطورة، فقد اقتصر الأمر على بيرو والفلبين في تطبيق البرامج الوطنية الملائمة. ويمكن في المستقبل تقديم أنظمة علاجية معجلة وتحصينات مستجدة لتبسيط عملية تقديم الخدمة ورفع مستوى الكفاءة في التكلفة.

**الاستنتاج:**

تراعي الوقاية السابقة على التعرض لداء السُعار عوامل الأمان والاستمناع ويُنصح بالالتفات إليها: (ا) إذا ما تعذرت سبل الحصول على الوقاية العلاجية بعد التعرض للمرض أو تأخرت، و(ب) إذا ما كانت مخاطر التعرض عالية ولا تلقى حقها من الاهتمام، و(جـ) إذا ما استعصت السيطرة على السُعار في ملاجئ الحيوانات. ولا ينبغي للوقاية السابقة على التعرض للمرض أن تنتقص من جهود تحصين الكلاب، أو الوقاية اللاحقة على التعرض له، أو التوعية لزيادة الوعي بداء السُعار في المجتمعات المحلية.

## 摘要

**目的:**

为了审查狂犬病暴露前预防（包括加速治疗，与其他疫苗联合施药及加强剂量）的安全性与免疫原性，以及其成本效益和可推荐使用性，尤其是在高发地区。

**方法:**

我们在美国国立医学图书馆 (PubMed)、国际农业和生物研究中心 (Centre for Agriculture and Biosciences International)、考科蓝图书馆 (Cochrane Library) 以及 Web of Science 数据库中查找了 2007 年至 2016 年 1 月 29 日期间刊发的与狂犬病暴露前预防相关的论文。此外，我们还回顾了秘鲁与菲律宾暴露前预防运动的现场数据。

**结果:**

狂犬病暴露前预防在儿童与成人人群中具有安全性和免疫原性，此外在与其他儿童常规疫苗接种及流行性乙型脑炎疫苗联合用药时也能发挥其安全性与免疫原性。 现有证据表明短期及少剂量方案具有安全性和免疫原性，其加强剂量间隔可延长至 10 年。 少数成本研究表明，多数情况下，当前疫苗及其配送成本以及暴露前预防活动的成本效益不高。 尽管早已提倡针对高危人群采用暴露前预防，但仅有秘鲁与菲律宾实施了适当的国家项目。 未来，快速治疗方案及新型疫苗将有可能简化配送流程并提高可承受性。

**结论:**

狂犬病暴露前预防具有安全性和免疫原性，应考虑： (i) 暴露后预防受限或滞后的地方；(ii) 暴露风险高且尚未发现的地方；以及 (iii) 控制狂犬病动物宿主困难的地方。 暴露前预防不应转移犬只疫苗的接种工作，此外还应提供接触后预防或教育以提高当地社区的狂犬病意识。

## Резюме

**Цель:**

Проанализировать безопасность и иммуногенность доконтактной профилактики бешенства (в том числе ускоренный курс вакцинации, назначение с другими вакцинами и ревакцинации), ее экономическую эффективность и рекомендации по применению, особенно в условиях высокого риска.

**Методы:**

Авторы осуществили поиск статей по доконтактной профилактике бешенства, опубликованных между 2007 годом и 29 января 2016 года, в базах данных PubMed, Международного центра по сельскому хозяйству и биологическим наукам (CABI), Кокрановской библиотеки и Web of Science. Авторы проанализировали данные, полученные в рабочих условиях в ходе кампаний по доконтактной профилактике бешенства в Перу и на Филиппинах.

**Результаты:**

Доконтактная профилактика бешенства была безопасной и способной вызывать иммунный ответ у детей и взрослых, в том числе в сочетании с плановой иммунизацией детей и вакцинацией против японского энцефалита. На основании доступных данных можно сделать вывод о том, что более короткие курсы и курсы с использованием меньшего количества доз безопасны, вызывают иммунный ответ и интервалы ревакцинации могут быть увеличены до 10 лет. Судя по результатам немногочисленных анализов расходов, при текущих затратах на вакцинацию применение имеющейся в настоящее время вакцины в кампаниях по доконтактной профилактике не было бы экономически эффективно в большинстве случаев. Хотя доконтактная профилактика рекомендуется для групп населения, подвергающихся высокому риску, соответствующие национальные программы были внедрены только в Перу и на Филиппинах. В будущем ускоренные курсы лечения и новые вакцины, возможно, позволят упростить выполнение вакцинации и повысить ее ценовую доступность.

**Вывод:**

Доконтактная профилактика бешенства безопасна, способна вызвать иммунный ответ и должна быть рекомендована: 1) когда доступ к постконтактной профилактике ограничен или предоставляется несвоевременно; 2) когда риск заражения велик или может быть не распознан; 3) когда борьба с бешенством у животных-носителей затруднительна. Доконтактная профилактика бешенства не должна быть поводом для отказа от мероприятий по вакцинации собак, проведения постконтактной профилактики или информационно-просветительской работы для привлечения внимания местного населения к проблеме бешенства.

## Introduction

Rabies is a preventable yet fatal disease that is responsible for approximately 59 000 deaths each year.^1^ However, widespread underreporting of rabies cases means that the actual number of deaths is likely to be higher. Poor and rural populations are disproportionately affected, with the majority of deaths occurring in children younger than 15 years in Asia and Africa.^2^ Ninety-nine per cent of human rabies cases result from dog bites and, once symptoms begin, the disease is almost invariably fatal.^3^ Human rabies is preventable through canine vaccination to eliminate rabies at its source or by administering rabies vaccines and immunoglobulin following bites, scratches or saliva exposure from suspected rabid mammals (i.e. postexposure prophylaxis).^4^

Another preventive strategy is pre-exposure prophylaxis, which involves giving a series of intramuscular or intradermal injections of rabies vaccine to prime the immune system. This enables fast recall of memory immune responses once a person is re-exposed to the virus.^4^ Moreover, people who have received pre-exposure prophylaxis require fewer doses of postexposure rabies vaccine and can be treated without rabies immunoglobulin, which is costly and difficult to procure.^4^ Although preventing rabies in dogs is the most cost-effective way of preventing human rabies deaths, pre-exposure prophylaxis is valuable for people at a high disease risk,^5^ particularly in areas where controlling disease in the animal reservoir is difficult or has not been implemented and in areas where access to postexposure prophylaxis and rabies immunoglobulin is unreliable or nonexistent. National pre-exposure prophylaxis programmes for high-risk populations have been implemented in Peru and the Philippines.^6^^,^^7^

In 2010, a World Health Organization (WHO) position paper on rabies vaccines called for studies on the feasibility, cost–effectiveness and long-term impact of incorporating vaccines derived from cell culture or embryonated eggs into immunization programmes for children where canine rabies is a public health problem.^5^ The paper also made recommendations on pre-exposure prophylaxis regimens and on the frequency of booster vaccinations and serological surveillance for at-risk individuals, such as veterinarians. The aim of this study was to review the scientific literature published between 2007 and 2016, as well as field data, to assess the current use and cost–effectiveness of pre-exposure rabies prophylaxis (excluding travel vaccines), particularly in children and in high-risk settings, in the context of recommendations made in the 2010 WHO rabies vaccine position paper on pre-exposure prophylaxis and booster vaccine administration.

## Methods

### Literature search

**Fig. 1:**
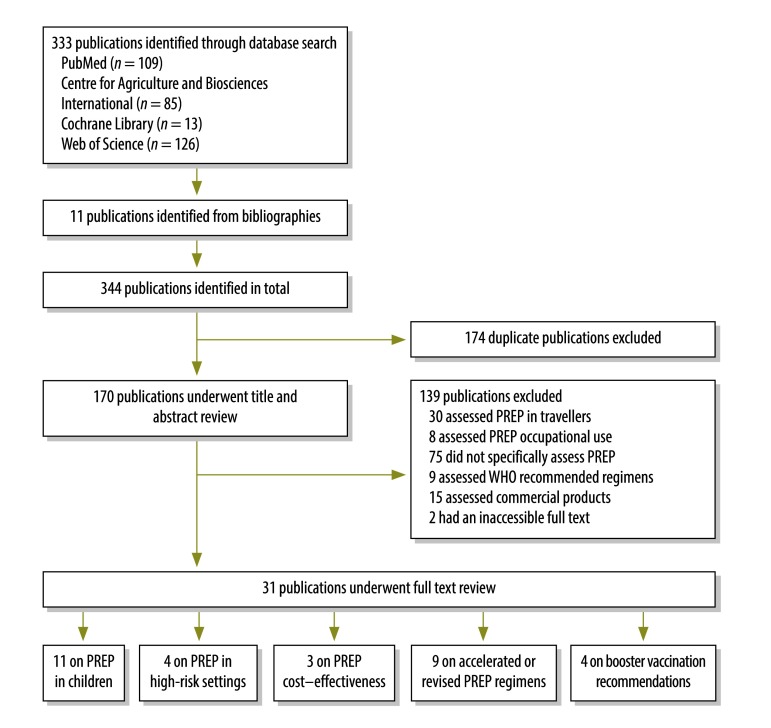
**Flowchart showing the selection of publications on pre-exposure rabies prophylaxis, 2007–2016**

PREP: pre-exposure prophylaxis; WHO: World Health Organization.

Our literature review was intended as an update of the review of the evidence on pre-exposure prophylaxis carried out for the 2010 WHO rabies vaccine position paper. Our search was conducted according to preferred reporting items for systematic reviews and meta-analyses guidelines.^8^ We searched the PubMed, Centre for Agriculture and Biosciences International, Cochrane Library and Web of Science databases for papers on pre-exposure rabies prophylaxis published between 2007 and 29 January 2016 (Fig. 1) using the search string: “rabies” AND “pre-exposure” AND (“prophylaxis” OR “vaccin*”). We started at 2007 to include studies published after completion of the review for the WHO position paper. Additional references were obtained from citations in relevant publications. We excluded studies that assessed: (i) postexposure prophylaxis only; or (ii) pre-exposure prophylaxis either occupationally or in travellers. As we considered the safety and immunogenicity of WHO-recommended vaccination regimens for pre-exposure prophylaxis to be well established, we also excluded papers that confirmed the efficacy of these regimens, unless they specifically assessed the safety and immunogenicity of pre-exposure prophylaxis in children or given in combination with other vaccines. We included any type of study, in any language and from any country that assessed: (i) pre-exposure prophylaxis in children; (ii) the cost–effectiveness of pre-exposure prophylaxis; (iii) accelerated or revised pre-exposure prophylaxis regimens; or (iv) booster vaccination recommendations. Studies that assessed the cost of pre-exposure prophylaxis and its use in children were included regardless of publication date.

### Field data

We reviewed field data from completed and ongoing pre-exposure prophylaxis campaigns in Peru and the Philippines. In Peru, the campaigns targeted people living in remote areas who were at risk of contracting rabies from vampire bats, whereas in the Philippines they targeted children at risk of dog-transmitted rabies.

#### Peru

In Peru, vampire bats are a common source of rabies: the life-time risk of a bat bite in rural Amazon basin populations is reported to be 41 to 88%.^9^^,^^10^ Outbreak reports and responses are delayed by the remoteness of these populations and controlling rabies in the bat reservoir is challenging.^11^ Following a rabies outbreak in 2011, Peru began a mass pre-exposure prophylaxis vaccination campaign that targeted people in Condorcanqui and Bagua Provinces at a high risk of rabies from vampire bats.^6^ The risk was regarded as high in these provinces because: (i) bat bites were common; (ii) there was evidence of rabies in circulation; (iii) housing conditions increased vulnerability; (iv) protective measures among the population were lacking; (v) tools for vector control were lacking; and (vi) the remote location of villages delayed health service responses. The campaign involved administering three intramuscular doses of human diploid cell or purified Vero cell vaccine on days 0, 7 and 28. Villages were prioritized to receive the intervention by classifying their epidemiological risk using the following variables: (i) the endemicity of bat rabies; (ii) the number of human rabies cases within the previous 6 months; (iii) the number of livestock rabies cases within the previous 6 months; (iv) the frequency of vampire bat bites (where there was bite surveillance); (v) history of postexposure prophylaxis; and (vi) important, recent ecological changes, such as an increase in the population, a change in the feeding habits of vampire bats, illegal mining or deforestation. Among villages with a history of postexposure prophylaxis interventions, priority was given to those in which the intervention took place more than 1 year previously, those where a low percentage of the population had received postexposure prophylaxis and those close to the site of a recent outbreak or of documented circulation of the rabies virus.

#### Philippines

In the Philippines, pre-exposure rabies prophylaxis is recommended as an additional intervention for high-risk individuals, such as children and people at occupational exposure. In 2007, the Philippine Government implemented a Department of Health recommendation that free routine pre-exposure prophylaxis should be provided for school children aged 5 to 14 years who are living in high-risk areas.^7^^,^^12^ To be included an area had to have: (i) an incidence of human and canine rabies above the  national average; (ii) an incidence of animal bites above the  national average; (iii) no or low canine vaccination coverage, which was defined as less than  30% coverage of the estimated dog population; and (iv) limited access to postexposure prophylaxis, for example, due to geographical isolation, inadequate treatment facilities or poverty. Schoolchildren were targeted because almost 50% of all rabies exposure in the Philippines occurs in children younger than 15 years. Child deaths due to rabies are associated with poverty and, where postexposure prophylaxis is available, with limited or delayed access to health services. The rationale for pre-exposure prophylaxis was that it: (i) may protect children who do not receive postexposure prophylaxis, for example, after unremarked exposure (i.e. if their antibody titre at exposure is ≥ 0.5 IU/mL); (ii) may protect patients when postexposure prophylaxis is delayed; (iii) accelerates antibody responses to postexposure prophylaxis; and (iv) reduces the cost of postexposure prophylaxis by removing the need for rabies immunoglobulin and reducing the number of postexposure prophylaxis doses required from 8 to 2 (Table 1). The pre-exposure prophylaxis schedule consisted of administering three intradermal doses of purified Vero cell or chick embryo cell vaccine on days 0, 7 and 28.

**Table 1:** Postexposure rabies prophylaxis regimens, by pre-exposure prophylaxis, the Philippines, 2007

Exposure category^a^	Following pre-exposure rabies prophylaxis			Without pre-exposure rabies prophylaxis	
	Rabies vaccine	Equine rabies immunoglobulin		Rabies vaccine	Equine rabies immunoglobulin
Category II	1 intradermal dose on days 0 and 3 (i.e. 2 doses)	No		2 intradermal doses on days 0, 3, 7 and 28 (i.e. 8 doses)	No
Category III	1 intradermal dose on days 0 and 3 (i.e. 2 doses)	No		2 intradermal doses on days 0, 3, 7 and 28 (i.e. 8 doses)	Yes, with the volume dependent on body weight

^a^ Category-II exposure is defined as nibbling of uncovered skin, minor scratches or abrasions without bleeding and category-III exposure, as single or multiple transdermal bites or scratches, contamination of mucous membranes with saliva from licks, licks on broken skin, exposures to bats.^5^

## Results

The systematic review of the literature identified 31 publications on pre-exposure rabies prophylaxis that met inclusion criteria (Table 2).

**Table 2:** Publications on pre-exposure rabies prophylaxis, systematic review of the literature, 2007–2016

Reference	Publication type	Publication date	Study location	Prophylaxis	Vaccinees
Aikimbayev et al.^13^	Meeting report	2014	Middle East, Eastern Europe, Central Asia	N/A	Children and adults
Banga et al.^14^	Journal article	2014	United States	N/A	Adults
Brown et al.^15^	Journal article	2011	United Kingdom	N/A	Adults
Brown et al.^16^	Journal article	2008	United Kingdom	N/A	Adults
Chulasugandha et al.^17^	Journal article	2006	Thailand	PVRV, PCECV	Children
Cunha et al.^18^	Journal article	2010	Brazil	PVRV, PCECV	Adults
Dodet et al.^19^	Meeting report	2009	Viet Nam	N/A	Children and adults
Dodet^7^	Meeting report	2010	Philippines	N/A	Children and adults
Hampson et al.^1^	Journal article	2015	Worldwide	N/A	Children and adults
Jelinek et al.^20^	Journal article	2015	Germany	PCECV, Japanese encephalitis vaccine	Adults
Kamoltham et al.^21^	Journal article	2011	Thailand	PVRV	Children
Kamoltham et al.^22^	Journal article	2007	Thailand	PCECV	Children
Khawplod et al.^23^	Journal article	2008	Thailand	PCECV, PVRV	Adults
Khawplod et al.^24^	Journal article	2012	Thailand	PCECV, PVRV	Adults
Khawplod et al.^25^	Journal article	2007	Thailand	PCECV, PVRV	Adults
Lang et al.^26^	Journal article	1997	Viet Nam	PVRV	Children
Lang et al.^27^	Journal article	2009	Viet Nam	PVRV combined with vaccination against diphtheria, tetanus, pertussis (whole-cell vaccine) and poliomyelitis (inactivated vaccine)	Children
Lau & Hohl^28^	Journal article	2013	Australia	PCECV	Children and adults
Lim & Barkham^29^	Journal article	2010	Singapore	PVRV	Adults
Liu^30^	Journal article	2012	Worldwide	N/A	Children
Lumbiganon et al.^31^	Journal article	1989	Thailand	PCECV	Children
Malerczyk et al.^32^	Journal article	2013	Germany	PCECV	Children
Mills et al.^33^	Journal article	2011	Australia	HDCV	Children and adults
Pengsaa et al.^34^	Journal article	2009	Thailand	PCECV combined with Japanese encephalitis vaccine	Children
Ravish et al.^35^	Journal article	2013	India	PCECV	Children
Shanbag et al.^36^	Journal article	2008	India	PVRV, PCECV	Children
Strady et al.^37^	Journal article	2009	France	HDCV, PVRV	Children
Sudarshan et al.^38^	Journal article	2011	Worldwide	N/A	Children and adults
Vashishtha et al.^39^	Journal article	2014	India	N/A	Children
Vien et al.^40^	Journal article	2008	Viet Nam	PVRV combined with vaccination against diphtheria, tetanus, pertussis (whole-cell vaccine) and poliomyelitis (inactivated vaccine)	Children
Wongsaroj et al.^41^	Journal article	2013	Thailand	PVRV	Adults

HDCV: human diploid cell vaccine; N/A: not available; PCECV: purified chick embryo cell vaccine; PVRV: purified Vero cell rabies vaccine.

### Safety and immunogenicity in children

#### Literature search

The search identified 11 studies on the safety and immunogenicity of pre-exposure prophylaxis in children aged 2 months to 15 years, including two published before 2007 (Table 3). All found it safe and immunogenic in both infants and children. Three found it safe and immunogenic for up to 5 years when given in combination with other childhood vaccines such as those against Japanese encephalitis, diphtheria, tetanus, pertussis and poliomyelitis (both oral and inactivated vaccines).^27^^,^^34^^,^^40^

**Table 3:** Pre-exposure rabies prophylaxis in children, systematic review of the literature, 1989–2016

Reference	Age group (years)	Vaccine	Vaccination route	Regimen	Antibody titre (IU/mL)^a^		Comments
					Primary response	Recall response	
Lang et al.,^27^ Vien et al.^40^ and Lang et al.^26^^b^	< 1	PVRV	Intramuscular	2 doses at 2 and 4 months of age	20.1	> 1 (assessed after 5 years)	Combined with vaccination against diphtheria, tetanus, pertussis and polio (inactivated vaccine)^27^^,^^40^
Pengsaa et al.^34^	1–1.5	PCECV	Intramuscular or intradermal	1 dose on days 0, 7 and 28; or 1 dose on days 0 and 28	15–41 (intramuscular); 4.1–8.5 (intradermal)	103–299 (intramuscular); 8.0–38 (intradermal) – both assessed after 1 year	Combined with vaccination against Japanese encephalitis: antibody titres were higher following intramuscular than intradermal administration
Lumbiganon et al.^31^^,^^b^	2–15	PCECV	Intramuscular or intradermal	1 dose on days 0, 7 and 28	4.7–47	ND	Antibody titres were higher following intramuscular than intradermal administration
Kamoltham et al.^21^ and Kamoltham et al.^22^	5–8	PCECV	Intradermal	1 dose on days 0, 7 and 28; or 1 dose on days 0 and 28	> 2	8.9–27.3 (assessed after ≥ 1 year)	All children had an antibody titre > 0.5 IU/mL within 14 days of the booster dose, regardless of the time interval and the number of doses initially received
Ravish et al.^35^	5–10	PCECV	Intradermal	1 dose on days 0, 7 and 21	ND	ND	80.4% of children completed treatment; there were no serious adverse reactions
Shanbag et al.^36^	6–13	PVRV or PCECV	Intramuscular	1 dose on days 0, 7 and 28	12.2–14.5	ND	None
Strady et al.^37^	12–79	HDCV or PVRV	Intramuscular	1 dose on days 0, 7 and 28; or 1 dose on days 0 and 28	0.1–48 (assessed after 1 year)	51 (3 doses); 13 (2 doses) – both assessed after 1 year	None
Malerczyk et al.^32^	< 15	PCECV	N/A	N/A	N/A	N/A	This review of > 1200 children treated over > 25 years concluded that the vaccine was safe and immunogenic, whether given intramuscularly or intradermally

HDCV: human diploid cell vaccine; N/A: not applicable; ND: not determined; PCECV: purified chick embryo cell vaccine; PVRV: purified Vero cell rabies vaccine.

^a^ The values are either geometric means or ranges, as appropriate.

^b^ Although this study was published before 2007, it has been included because the results are still relevant.

### High-risk settings

#### Literature search

Pre-exposure prophylaxis programmes for high-risk populations, and especially children, were strongly recommended in reports of expert meetings on rabies and child health in Asia and the Middle East.^6^^,^^13^^,^^19^ In India, the Academy of Pediatrics called for its inclusion in the immunization schedule for high-risk children younger than 18 years.^39^

#### Peru

**Fig. 2:**
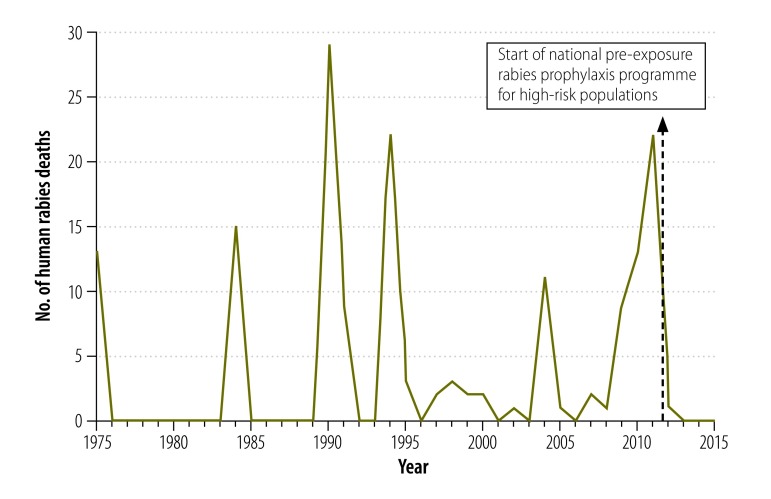
**Human rabies deaths, Amazonas Region, Peru, 1975–2015**

Notes: The Amazonas Region is the political division of Peru that includes Condorcanqui and Bagua Provinces, not the Amazon ecological region. All human rabies deaths reported in the figure occurred in Bagua and Condorcanqui Provinces.

In 2011, pre-exposure prophylaxis was administered in 286 localities in the Amazonas Region: 86% were in Condorcanqui Province and 14% were in Bagua Province. In total, 13 986 people were immunized. In these areas, the number of rabies deaths dropped from 13 in 2010 and 20 in 2011 to zero child deaths and only two adult deaths (both had refused vaccination) in 2012.^6^ Fig. 2 shows the number of human rabies deaths in Bagua and Condorcanqui Provinces between 1975 and 2015. The number of reported bat bites, which is used as a surrogate for rabies exposure, decreased between 2010 and 2013 (Table 4) but there was no change in the risk factors for bites, such as the number of houses bats could enter. In areas adjacent to the Amazonas Region, in which pre-exposure prophylaxis was not implemented, there were outbreaks of human rabies in 2011, 2013 and 2015 (unpublished data, 2015). The programme was extended until 2015 to cover an additional 423 communities in Bagua and Condorcanqui Provinces at a high risk of rabies. By 2015, 71 400 people in the Amazonas Region (i.e. 86% of the population) had received pre-exposure prophylaxis and, by the end of 2014, 121 285 people (i.e. 76% of the target population) in Cusco, Junín and Loreto Regions had also received it. No serious adverse events were reported.

**Table 4:** Bat bites by region, Peru, 2009–2013**^6^**

Region	No. of bat bites reported						% of all reported bat bites
	2009	2010	2011	2012	2013	Total	
Amazonas	1 576	5 714	2 145	1 733	833	12 001	59.2
Cusco	50	169	36	441	20	716	3.5
Loreto	1 122	856	1 458	1 380	590	5 406	26.7
Junin	119	415	179	142	29	884	4.4
Others	465	224	295	229	41	1 254	6.2
All	3 332	7 378	4 113	3 925	1 513	20 261	100.0

#### Philippines

By April 2010, the routine pre-exposure prophylaxis immunization programme had achieved an average coverage of 47.25% in the target population: 21 637 high-risk children in 31 schools in seven regions were immunized (unpublished data, 2010). In the town of Cabusao, 188 schoolchildren received at least one vaccine dose (i.e. 86% of those eligible) and 90% of the 188 completed the pre-exposure prophylaxis regimen. Subsequently, 3.5% received postexposure prophylaxis within 3 years following suspected exposure. The programme was stopped in 2011 because a large increase in rabies exposure led to a vaccine shortage and priority was given to the immunization of people involved in canine vaccination campaigns. Pre-exposure prophylaxis of schoolchildren was planned to restart in 2016.

### Cost–effectiveness

#### Literature search

Few recent studies have assessed the cost–effectiveness of pre-exposure rabies prophylaxis. One study estimated the annual global direct cost of administering postexposure rabies prophylaxis at 1.7 billion United States dollars (US$), plus an additional US$ 1.3 billion in lost income.^1^ In a cost assessment of pre-exposure prophylaxis, researchers showed that it would be cost-neutral if 1% of children were exposed to rabies each year and if the price of the vaccine did not exceed US$ 1.32 per dose, once the cost of postexposure prophylaxis boosters required after exposure was taken into account.^30^ The acceptable vaccine cost increased in proportion to the incidence of rabies. In a Thai study, the estimated cost of pre-exposure prophylaxis for children ranged from US$ 2.00 to 7.25 per child depending on the schedule and vaccine used: there was an additional cost of US$ 18.00 to 23.50 per child if postexposure prophylaxis was required later.^17^ Pre-exposure prophylaxis became cost-comparable to the least expensive postexposure schedule (i.e. intradermal immunization without rabies immunoglobulin) when the annual risk of a dog bite was approximately 23%. If equine or human rabies immunoglobulin was used with postexposure vaccines, pre-exposure prophylaxis was cost-comparable when the annual risk of a dog bite was 7% or 3%, respectively. As over 30% of Thai children had been bitten by a dog by the age of 15 years, it was estimated that the actual incidence of dog bites in the population of central Thailand was only 2.3% per year. Consequently, pre-exposure prophylaxis with currently licensed vaccines would not be cost-effective in this setting.

#### Peru

The cost of the mass pre-exposure prophylaxis campaign was estimated to be US$ 4 111 000, of which US$ 3 560 000 was the cost of the vaccine. The average cost per immunized person was US$ 69. By assuming that the risk of rabies was constant (i.e. the rabies virus remained in circulation and the risk of a bat bite was unchanged) and that, each year, rabies caused 20 deaths per 50 000 people in Condorcanqui Province without pre-exposure prophylaxis, we estimated the cost of pre-exposure prophylaxis to be US$ 205 000 per life saved after the first year.^6^ After 5 years, the cost decreased to US$ 41 000 per life saved. This amount is comparable to the cost of treating one rabies-infected individual, including the cost of transport, laboratory diagnosis and hospitalization. The use of intradermal vaccinations would reduce the vaccination cost by 80%. However, intramuscular vaccination continues to be used in Peru because: (i) there is no shortage of rabies vaccine in the country; (ii) staff have not been trained in the multiple uses of rabies vaccine vials for intradermal administration; and (iii) the national authorities elected to use the intramuscular route to minimize the risk of errors in vaccine administration.

#### Philippines

Pre-exposure prophylaxis with three doses of purified Vero cell or chick embryo cell vaccine was estimated to cost US$ 4.77 per patient (unpublished data, 2015; (Table 5). For a patient weighing between 26 and 50 kg, pre-exposure prophylaxis reduced the cost of postexposure prophylaxis by up to 38% following category-II exposure (i.e. “nibbling of uncovered skin, minor scratches or abrasions without bleeding”)^5^ and by up to 85% following category-III exposure (i.e. “single or multiple transdermal bites or scratches, contamination of mucous membranes with saliva from licks, licks on broken skin, exposures to bats”),^5^ after the cost of pre-exposure prophylaxis was taken into account.

**Table 5:** Cost of postexposure rabies prophylaxis, the Philippines, 2007

Exposure category^a^	Cost in US$ per patient (treatment specifics)			Savings per patient (weight range: 26–50 kg) who had pre-exposure prophylaxis	
	Patients who had pre-exposure prophylaxis	Patients (weight range: 26–50 kg) who did not have pre-exposure prophylaxis		US$	(%)
Category II	3.19 (2 intradermal doses of PCECV or PVRV at US$ 1.59 per dose; no RIG)	12.76 (8 intradermal doses of PCECV or PVRV at US$ 1.59 per dose; no RIG)		4.80	38
Category III	3.19 (2 intradermal doses of PCECV or PVRV at US$ 1.59 per dose; no RIG)	51.76 (8 intradermal doses PCECV or PVRV at US$ 1.59 per dose; 2 vials of ERIG at US$ 19.52 per vial)		43.80	85

ERIG: equine rabies immunoglobulin; PCECV: purified chick embryo cell vaccine; PVRV: purified Vero cell rabies vaccine; RIG: rabies immunoglobulin; US$: United States dollar.

^a^ Category-II exposure is defined as nibbling of uncovered skin, minor scratches or abrasions without bleeding and category-III exposure, as single or multiple transdermal bites or scratches, contamination of mucous membranes with saliva from licks, licks on broken skin, exposures to bats.^5^

Notes: The percentage saving is the cost saving divided by the cost of postexposure prophylaxis in a patient who did not have pre-exposure prophylaxis × 100. Prices were converted at a rate of US$ 1 per 47.65 Philippine pesos. The cost of postexposure rabies prophylaxis was the cost at bite centres taking part in a national pre-exposure rabies prophylaxis programme for high-risk populations, which was lower than in hospitals and private bite centres. The cost of pre-exposure rabies prophylaxis was US$ 4.77 per patient (US$ 1.59 per intradermal dose × 3). In calculating savings, the cost of pre-exposure prophylaxis was taken into account.

### Accelerated or revised regimens

Nine studies investigated the safety and immunogenicity of an accelerated or revised pre-exposure prophylaxis regimen (Table 6; available at http://www.who.int/bulletin/volume/95/03/16-173039). Administering all vaccine doses within 1 week^22^^,^^23^^,^^28^^,^^33^ or in one^23^^,^^24^ or two visits^41^ elicited an adequate antibody titre of 0.5 IU/mL or higher for up to 1 year,^23^^–^^25^^,^^28^^,^^33^ even when given in combination with Japanese encephalitis vaccine.^20^ Adequate titres were observed in people who received a total dose of at least 2 IU of intradermal pre-exposure prophylaxis.^15^ Factors associated with an inadequate antibody titre included: (i) a period of more than 21 days between the first and third doses; (ii) male sex; (iii) vaccine type or manufacturer; and (iv) a body mass index of 25 kg/m^2^ or higher.^14^

**Table 6:** Accelerated or revised pre-exposure rabies prophylaxis, systematic review of the literature, 2007–2016

Reference	Study type	No. of study participants	Vaccine	Vaccination route	Regimen	Antibody titre (IU/mL)		Comments
						Primary response	After booster vaccination	
Kamoltham et al.^22^	Randomized, open-label phase-II clinical trial	703	PCECV	Intradermal	(i) 0.1 mL on days 0 and 28; and (ii) 0.1 mL on days 0, 7 and 28	ND	(i) 10.76 (GMT; range: 1.87–37); and (ii) 22.12 (GMT; range: 2.13–199) – both measured 14 days after receiving 0.1 mL PCECV booster vaccination on days 365 and 368	Seroconversion^a^ occurred within 14 days of booster vaccination in all vaccinees who received two or three doses of pre-exposure prophylaxis
Khawplod et al.^25^ and Khawplod et al.^23^	Randomized, prospective	96 and 52	PVRV and PCECV	Intradermal and intramuscular	(i) 0.1 mL PVRV intradermally at two sites on days 0, 7 and 28; (ii) 0.1 mL PVRV intradermally at two sites on days 0, 3 and 7; (iii) 1.0 mL PVRV intramuscularly at one site on days 0, 3 and 7; (iv) 0.1 mL PVRV intradermally at two sites on day 0; (v) 0.1 mL PVRV intradermally at two sites on days 0, 3 and 7 and at one site on days 28 and 90; and (vi) 0.1 mL PCECV intradermally at two sites on days 0, 3 and 7 and at one site on days 28 and 90	(i) 0.96 (GMT) on day 360; (ii) 1.12 (GMT) on day 360; (iii) 0.97 (GMT) on day 360; (iv) 0.41 (GMT) on day 360; (v) 5.84 (GMT) on day 28; and (vi) 5.96 (GMT) on day 28	(i) 49.39 (GMT) on day 374; (ii) 105.08 (GMT) on day 374; (iii) 125.00 (GMT) on day 374; (iv) 51.96 (GMT) on day 374; (v) ND; and (vi) ND – all measured after booster vaccination with 0.1 mL PVRV intradermally at two sites on days 360 and 363	Seroconversion^a^ occurred after booster vaccination with all regimens; the two studies used the same regimens and reported the same data
Mills et al.^33^	Case series	420	HDCV	Intradermal	0.1 mL at two sites on days 0 and 7	> 0.5 in 94.5% of vaccinees on day 28	ND	Seroconversion^a^ occurred in 94.5% of vaccinees by day 28 following a two-visit pre-exposure prophylaxis regimen
Khawplod et al.^24^	Abbreviated, prospective	109	PCECV	Intradermal and intramuscular	(i) 0.1 mL intradermally on days 0, 7 and 21, followed by a 1.0-mL intramuscular booster on days 360 and 363; (ii) 0.1 mL intradermally on days 0, 7 and 21, followed by a 0.1-mL intradermal booster at four sites on day 360; (iii) 0.1 mL intradermally at two sites on day 0, followed by a 1.0-mL intramuscular booster on days 360 and 363; (iv) 0.1 mL intradermally at two sites on day 0, followed by a 0.1-mL intradermal booster at four sites on day 360; (v) 1.0 mL intramuscularly on day 0, followed by a 1.0-mL intramuscular booster on days 360 and 363; and (vi) 1.0 mL intramuscularly on day 0, followed by a 0.1-mL intradermal booster at four sites on day 360	(i) 0.49 (NAb); (ii) 0.30 (NAb); (iii) 0.15 (NAb); (iv) 0.10 (NAb); (v) 0.08 (NAb); and (vi) 0.11 (NAb) – all measured before booster vaccination on day 360	(i) 11.27 (NAb); (ii) 42.49 (NAb); (iii) 9.71 (NAb); (iv) 11.96 (NAb); (vi) 10.13 (NAb); and (vi) 13.33 (NAb) – all measured 7 days after booster vaccination	Seroconversion^a^ occurred within 7 days of booster vaccination for all regimens assessed
Lau & Hohl^28^	Case series	54	PCECV	Intradermal	0.1 mL at two sites on days 0 and 7	> 0.5 in 94.4% of vaccinees on day 28	ND	Seroconversion^a^ occurred in 94.4% of vaccinees by day 28
Wongsaroj et al.^41^	Randomized, prospective	55	PVRV	Intradermal and intramuscular	(i) 0.1 mL intradermally at two sites on days 0 and 21; and (ii) 0.5 mL intramuscularly on days 0, 7 and 21	(i) 4.51 (NAb); and (ii) 6.74 (NAb) – both measured on day 35	(i) 14.38 (GMT); and (ii) 14.06 (GMT) – both measured 14 days after booster vaccination with 0.1 mL PVRV intradermally on days 360 and 363	Seroconversion^a^ occurred within 14 days of booster vaccination with both regimens
Jelinek^20^	Randomized, observer-blinded, multicentre	661	PCECV	Intramuscular	(i) 1.0 mL on days 0, 7 and 28, with standard Japanese encephalitis vaccine regimen; (ii) 1.0 mL on days 0, 3 and 7, with accelerated Japanese encephalitis vaccine regimen; and (iii) 1.0 mL PCECV alone on days 0, 7 and 28	> 0.5 in 97–100% of vaccinees on day 57	ND	Seroconversion^a^ occurred in 97–100% of vaccinees by day 57
Brown et al.^15^	Cohort study	12	PVRV (booster dose)	Intradermal	People with an antibody titre < 0.5 IU/mL following initial pre-exposure prophylaxis received one booster dose after 2 years to give a total vaccine dose ≥ 2 IU	0.18 (mean) before booster	17.33 (mean) after booster	Seroconversion^a^ occurred in all vaccinees who received ≥ 2 IU of vaccine

HDCV: human diploid cell vaccine; GMT: geometric mean titre; NAb: neutralizing antibody; ND: not determined; PCECV: purified chick embryo cell vaccine; PVRV: purified Vero cell rabies vaccine.

^a^ Seroconversion was defined as an antibody titre > 0.5 IU/mL.

### Booster vaccinations

Four studies investigated recommendations on booster vaccines (Table 7; available at http://www.who.int/bulletin/volume/95/03/16-173039). They concluded that: (i) the interval between booster vaccinations could be extended by up to 10 years;^16^ (ii) serological surveillance or booster vaccination after 1 year is advisable for people in high-risk occupations;^29^ (iii) serological testing after the third intramuscular or intradermal pre-exposure prophylaxis dose is unnecessary;^18^ and (iv) healthy subjects may not require postexposure prophylaxis boosters on re-exposure to rabies for up to 3 months after pre-exposure or previous postexposure prophylaxis.^38^

**Table 7:** Booster rabies vaccination recommendations, systematic review of the literature, 2007–2016

Reference	Study type	No. of participants	Vaccination regimen	Antibody titre (IU/mL)	Conclusion
Brown et al.^16^	Retrospective cohort study	89	Intradermal pre-exposure prophylaxis	≥ 0.5 after a mean of 5 years (range: 1–12) in 100% of vaccinees who received ≥ 0.6 mL of vaccine over two or three visits	Intradermal pre-exposure prophylaxis with 0.6 mL of vaccine over three visits could extend the interval before booster vaccination to 10 years
Lim & Barkham,^29^ Cohort 1	Retrospective cohort study	66	Three doses of PVRV pre-exposure prophylaxis	> 0.5 in 60.6% of vaccinees after 1 year	Serological surveillance or a booster vaccination 1 year after primary pre-exposure prophylaxis is advised for people in high-risk occupations
Lim & Barkham,^29^ Cohort 2	Retrospective cohort study	15	Four doses: three of pre-exposure prophylaxis and one booster dose given after a median of 10 years (range: 3–18)	> 0.5 in 100% of vaccinees after a median of 10 years (range: 3–18)	Serological surveillance or a booster vaccination 1 year after primary pre-exposure prophylaxis is advised for people in high-risk occupations
Cunha et al.,^18^ Group 1	Randomized controlled study	65	Intradermal pre-exposure prophylaxis	> 0.5 in 97% of vaccinees after a mean of 10 days and > 0.5 in 20–25% after a mean of 180 days	Serological testing after the third dose of pre-exposure prophylaxis is unnecessary^a^
Cunha et al.,^18^ Group 2	Randomized controlled study	62	Intramuscular pre-exposure prophylaxis	> 0.5 in 100% of vaccinees after a mean of 10 days and > 0.5 in 63–65% after a mean of 180 days	Serological testing after the third dose of pre-exposure prophylaxis is unnecessary^a^
Sudarshan et al.,^38^ Group 1	Literature review	577	Pre-exposure prophylaxis	> 0.5 in 100% after a mean of 3 months	It may be safe not to administer postexposure prophylaxis in healthy individuals re-exposed to rabies within 3 months of pre-exposure or previous postexposure prophylaxis
Sudarshan et al.,^38^ Group 2	Literature review	2795	Postexposure prophylaxis	> 0.5 in 99.9% after a mean of 3 months	It may be safe not to administer postexposure prophylaxis in healthy individuals re-exposed to rabies within 3 months of pre-exposure or previous postexposure prophylaxis

PVRV: purified Vero cell rabies vaccine.

^a^ This study did not follow up study participants 1 year after pre-exposure prophylaxis or simulate responses to postexposure prophylaxis.

## Discussion

Several studies demonstrated that pre-exposure rabies prophylaxis was safe and immunogenic in children and could be co-administered with other childhood vaccines.^21^^,^^22^^,^^27^^,^^32^^,^^34^^–^^37^^,^^40^ In addition, it could be given with the Japanese encephalitis vaccine in both adults and children. In most African countries pre-exposure rabies prophylaxis is unlikely to be included in the expanded programme on immunization because of competing priorities and because postexposure prophylaxis would still be required following suspected contact. Nevertheless, expert consultations advocate vaccination for people in remote, high-risk areas^6^^,^^19^^,^^36^ and national pre-exposure prophylaxis programmes have been implemented in Peru and the Philippines.^6^^,^^7^ In Peru, the programme was successful in preventing child deaths due to bat rabies in high-risk areas, which demonstrates the value of targeted pre-exposure prophylaxis in places where controlling disease in the animal reservoir is challenging.^11^ Although it can be difficult for individuals to recall the date of pre-exposure prophylaxis, this does not undermine its usefulness for saving human lives in situations where exposure is uncertain or there is limited access to biologicals. Vaccination certificates are often treasured and kept safe and, in Peru, the identification and recording of vaccinated individuals has improved nationally.

Pre-exposure rabies prophylaxis is also associated with cost savings because fewer postexposure vaccinations and no rabies immunoglobulin are required following suspected exposure. However, the few studies that assessed costs suggest that community vaccination at current vaccine and delivery costs would not be cost-effective in most situations.^1^^,^^17^^,^^30^^,^^42^^,^^43^ Preliminary studies on accelerated or revised regimens indicate that 1-week or even single-day regimens may be as effective as the recommended 3- to 4-week regimen: shorter treatment and fewer doses would make treatment simpler and less expensive.

The development of a more immunogenic rabies vaccine that provides life-long immunological memory with a single dose and that can be preserved at ambient temperatures, thereby eliminating the need for a cold chain, would make pre-exposure prophylaxis simpler and more cost-effective. The ideal vaccine would induce an antibody titre that remained above 0.5 IU/mL for decades and would protect people who fail to receive prompt booster immunization following exposure. In animal studies, attempts have been made to increase the current vaccine’s immunogenicity using adjuvants,^44^^,^^45^ genetic manipulation,^46^ adenovirus vectors derived from chimpanzee viruses^47^ and attenuated measles viruses,^48^^,^^49^ which would enable combined early childhood immunization against both measles and rabies.

Although pre-exposure prophylaxis using currently available biologicals may not be cost-effective in general, we believe it could be beneficial in: (i) remote communities where access to postexposure prophylaxis and rabies immunoglobulin is often delayed or nonexistent; (ii) situations in which the risk of exposure is high and may go unrecognized, for example, in young children or people exposed occupationally, such as veterinarians; or (iii) places where controlling rabies in the animal reservoir is difficult and the risk of human exposure is high, such as in the Amazon basin where bat rabies is endemic. It is important that staff involved in canine rabies control receive pre-exposure prophylaxis because of their higher risk of exposure. Currently, serological surveillance and booster vaccinations are recommended only for people at an occupational risk.^5^ Controlling canine rabies remains the cornerstone of preventing human rabies deaths. Pre-exposure prophylaxis should not distract from canine vaccination efforts, the provision of postexposure prophylaxis and education to raise local awareness of rabies. In high-risk areas, pre-exposure prophylaxis should be included in the expanded programme on immunization in children from 1 year of age,^5^ followed by a booster after 1 year. Vaccination should be documented with a certificate and any available medical records should be updated. Targeted, mass campaigns in remote, high-incidence areas should be considered to provide protection for both children and adults and travel recommendations should be provided for newcomers. Accelerated vaccination regimes and novel vaccines that provide life-long immunity with a single dose and are stable at ambient temperatures would make pre-exposure prophylaxis more cost-effective and easier to implement.
